# Supplementary material for: Clinical Significance of a 16S-rDNA Analysis of Heart Valves in Patients with Infective Endocarditis: a Retrospective Study
Source: Microbiol Spectr. 2023 May 17;11(3):e01136-23. doi: 10.1128/spectrum.01136-23 (PMC10269717; doi:10.1128/spectrum.01136-23)
Supplement: Supplemental file 1 — Supplemental material. Download spectrum.01136-23-s0001.docx, DOCX file, 0.02 MB [file spectrum.01136-23-s0001.docx]

**Appendix 1.** Overview of microbiological findings in episodes excluded due valve cultures or 16S-analysis not being performed.

**16S-analysis not taken** n= 66

| **Blood culture** | **Valve culture** | **16S-analysis** |
| --- | --- | --- |
| *S. aureus* | Negative | *-* |
| *S. aureus* | Negative | *-* |
| *S. aureus* | *S. aureus* | *-* |
| *S. lugdunensis* | *S. lugdunensis* | - |
| *K. pneumoniae* | Negative | *-* |
| *S. mitis* | *Acinetobacter* spp. | *-* |
| *E. faecalis* | Negative | *-* |
| *S. bovis* | Negative | *-* |
| *A. defectiva* | Negative | *-* |
| *S. mitis* | Negative | *-* |
| *S. mitis* | Negative | *-* |
| *E. faecalis* | Negative | *-* |
| *S. aureus* | Negative | *-* |
| *S. aureus* | Negative | *-* |
| *S. aureus* | Negative | *-* |
| *S. aureus* | Negative | *-* |
| *A. urinae* | Negative | *-* |
| *E. faecalis* | *E. faecalis* | *-* |
| *S. mitis* | Negative | *-* |
| *S. mitis* | Negative | *-* |
| *S. mitis* | Negative | *-* |
| *E. faecalis* | Negative | *-* |
| *S. agalactiae* | Negative | *-* |
| *S. aureus* | Negative | *-* |
| *S. aureus* | Negative | *-* |
| *S. aureus* | Negative | *-* |
| *S. mitis* | Negative | *-* |
| *S. mutans* | Negative | *-* |
| *S. mitis* | Negative | *-* |
| *S. bovis* | Negative | *-* |
| *S. bovis* | Negative | *-* |
| *E. faecalis* | *E. faecalis* | *-* |
| *S. mitis* | Negative | *-* |
| *C. glabrata* | *C. glabrata* | *- ** |
| *S. anginosus* | Negative | *-* |
| *S. mitis* | Negative | *-* |
| *S. epidermidis* | *S. epidermidis* | *-* |
| *S. mitis* | Negative | *-* |
| *E. faecalis* | *E. faecalis* | *-* |
| *E. faecalis* | Negative | *-* |
| *E. faecalis* | *E. faecalis* | *-* |
| *S. epidermidis* | *S. epidermidis* | *-* |
| *Gemella* spp. | *Gemella* spp. | *-* |
| *Gemella* spp. | Negative | *-* |
| *S. lugdunensis* | *S. lugdunensis* | *-* |
| *S. vestibularis* | Negative | *-* |
| *C. parapsilosis* | *C. parapsilosis* | *- *** |
| *S. anginosus* | Negative | *-* |
| Not taken | Negative | *-* |
| *S. mitis* | Negative | *-* |
| *E. faecalis* | *E. faecalis* | *-* |
| *S. aureus* | Negative | *-* |
| *S. mitis* | *S. mitis* | *-* |
| *S. aureus + S. agalactiae* | *S. aureus* | *-* |
| *S. aureus* | *S. aureus* | *-* |
| *S. aureus* | *S. aureus* | *-* |
| *S. aureus* | Negative | *-* |
| Negative | *Neisseria* spp. | *-* |
| *Streptococcus* Group G | Negative | *-* |
| Negative | Negative | *-* |
| *S. agalactiae* | Negative | *-* |
| *S. aureus* | Negative | *-* |
| *G. morbillorum* | Negative | *-* |
| *S. mitis* | Negative | *-* |
| *S. aureus* | Negative | *-* |
| *E. faecalis* | Negative | *-* |

**Valve culture not taken** n= 3

| *S. mitis* | *-* | *S. mitis.* |
| --- | --- | --- |
| *S. sanguinis* | *-* | *S. sanguinis* |
| *S. aureus* | - | Negative |

*18S-rDNA analysis not performed.

***C. parapsilosis* found in 18S-rDNA analysis.

**Appendix 2.** Overview of episodes with valve analyses defined as contaminated.

**Valve cultures defined as contaminated** n= 7

| **Blood culture** | **Valve culture** | **16S-analysis** |
| --- | --- | --- |
| *H. parainfluenzae* | *Acinetobacter* spp*.* | *H. parainfluenzae* |
| *S. haemolyticus* | *Acinetobacter* spp*.* | *S. haemolyticus* |
| *S. sanguinis* | Environmental bacteria | *S. sanguinis* |
| *S. epidermidis* | *C. acnes* | CoNS |
| *E. faecalis* | *C. acnes* | *E. faecalis* |
| *S. mitis + P. aeruginosa* | *C. parapsilosis* | *S. mitis + P. aeruginosa* |
| *S. gordonii* | *P. oryzihabitans* | *S. gordonii* |

**16S-analysis defined as contaminated** n= 2

| *C. albicans* | *C. albicans* | *Veillonella* spp*.* * |
| --- | --- | --- |
| *S. aureus* | Negative | Gram-negative environmental bacteria |

*18S-rDNA analysis not performed.

CoNS= Coagulase negative staphylococci

*Appendix 3.* Bacterial classification as labelled by the microbiological laboratory

|  | Blood cultures | Valve cultures | 16S-analysis |
| --- | --- | --- | --- |
| Viridans streptococci | 71 | 5 | 57 |
| *Streptococcus mitis* | 35 | 5 | 26 |
| *S. sanguinis* | 11 | 0 | 13 |
| *S. anginous* | 8 | 0 | 5 |
| *S. salivaruis* | 7 | 0 | 4 |
| *S. mutans* | 5 | 0 | 5 |
| *S. gordonii* | 3 | 0 | 1 |
| *S. parasanguinis* | 1 | 0 | 0 |
| *S. oralis* | 1 | 0 | 0 |
| α-hemolytic streptococci | 0 | 0 | 1 |
| *Streptococcus* spp. | 0 | 0 | 2 |
| Enterococci | 25 | 8 | 20 |
| *Enterococcus faecalis* | 24 | 8 | 19 |
| *E. faecium* | 1 | 0 | 1 |
| CoNS | 25 | 8 | 16 |
| *Staphylococcus epidermidis* | 19 | 6 | 3 |
| *S. lugdunensis* | 2 | 0 | 0 |
| *S. haemolyticus* | 1 | 0 | 1 |
| *S. waneri* | 1 | 1 | 1 |
| *S. caprae* | 1 | 0 | 0 |
| CoNS | 1 | 1 | 11 |
| HACEK-group | 10 | 0 | 10 |
| *Haemophilus parainfluenzae* | 6 | 0 | 7 |
| *Cardiobacterium* spp. | 2 | 0 | 1 |
| *C. hominis* | 0 | 0 | 1 |
| *Aggregatibacter* spp. | 1 | 0 | 1 |
| *A. aphrophilius* | 1 | 0 | 0 |
| *S. bovis* | 13 | 2 | 11 |
| *S. bovis* | 12 | 2 | 11 |
| *S. gallolyticus* | 1 | 0 | 0 |
| β-hemolytic streptococci | 17 | 3 | 19 |
| *S. agalactiae* | 12 | 2 | 12 |
| *S. dysgalactiae* | 4 | 0 | 6 |
| *S. pyogenes* | 1 | 1 | 1 |
|  |  |  |  |
